# Cone-beam computed tomographic analysis of maxillary sinus septa among Yemeni population: a cross-sectional study

**DOI:** 10.1186/s12903-023-03124-6

**Published:** 2023-07-08

**Authors:** Bassam A. Altayar, Barakat Al-Tayar, Weimin Lin, Saddam N. Al-wesabi, Eissa A. Al-shujaa, Karim Sakran, Quan Yuan, Mingyue Lyu

**Affiliations:** 1 State Key Laboratory of Oral Diseases & National Clinical Research Center for Oral Diseases, West China School of Stomatology, Sichuan University, Chengdu, 610041 China; 2 State Key Laboratory of Oral Diseases & National Clinical Research Center for Oral Diseases, Department of Oral Implantology, West China School of Stomatology, Sichuan University, Chengdu, China; 3 Dentistry Division, Faculty of Medicine and Health Sciences, Taiz University, Taiz, Yemen; 4 State Key Laboratory of Oral Diseases & National Clinical Research Centre for Oral Diseases, Department of Oral and Maxillofacial Surgery, West China School of Stomatology, Sichuan University, Chengdu, China

**Keywords:** CBCT, Maxillary sinus septa, Sinus membrane pathology, Sinus floor elevation, Dental implantation

## Abstract

**Background:**

Maxillary sinus septa increase perforation risk of Schneiderian membrane during the sinus floor elevation (SFE). Cone Beam Computed Tomography (CBCT) allows for a more precise assessment of the septal position; thus, preoperative CBCT analysis is substantial to avoid possible complications. This study aims to investigate the 3D characteristics of the maxillary sinus septa based on CBCT images. To our knowledge, no study reported the CBCT-based investigation for the sinus septa among Yemeni population.

**Materials and methods:**

This is a retrospective cross-sectional analysis of 880 sinus CBCT images 440 patients. The septa prevalence, locations, orientations, morphology, and associated factors were analyzed. The effect of age, gender, and dental status on the sinus septa and the relationship between sinus membrane pathology and sinus septa were also analyzed. Anatomage (Invivo version 6) was used for CBCT images analysis. Descriptive and analytical statistics were performed, and a *P*-value < 0.05 was significantly considered.

**Results:**

The maxillary sinus septa were found among 63.9% of patients and 47% of sinuses. The average septa height was 5.2 mm. 15.7% of patients had septa in the right maxilla, 18% in the left, and 30.2% in both. Gender, age, and dental condition had no influence on the presence of septa, and septa presence did not influence sinus membrane pathology. Many septa originated from the floor (54.5%), located in the middle (43%), with coronal orientation (66%) and complete configuration (58.2%).

**Conclusion:**

Based on our findings, the septa prevalence, locations, orientations, and morphology were significant and equivalent to the highest recorded in the literature yet. Thus, when sinus floor elevation is planned, CBCT imaging of the maxillary sinus is recommended for safe dental implantation.

**Supplementary Information:**

The online version contains supplementary material available at 10.1186/s12903-023-03124-6.

## Introduction

As the largest paranasal sinus, the maxillary sinus plays an essential role in pronunciation and resonance, reduces the skull weight, protects the skull base from trauma, and humidifies and warms the inhaled air [1]. The maxillary sinus has a pyramidal form, of which the base lies in the lateral wall of the nasal cavity and points towards the zygomatic process of the maxilla [2]. Maxillary sinus septa are cortical bone barriers or projections that develop from any interior wall of the sinus, first identified by Underwood in 1910; thus, it is also known as the Underwood septum [3].

Rehabilitation of patients with endosseous implants in the posterior maxilla may be hindered by pneumatization of the maxillary sinus and/or ridge atrophy. Sinus floor elevation (SFE) is one of the most reliable surgical techniques proposed to increase the bone volume for optimal implant placement [4, 5]. Nonetheless, it is a sensitive procedure that demands the surgical competence of the surgeons [6]. The most prevalent intraoperative complication is perforation of the Schneiderian membrane during SFE, which occurs in 11–56% of sinus floor elevation procedures [7, 8] that might lead to graft migration and sinus infection [2, 5]. Establishing an adequate surgical strategy and preventing intraoperative complications need detailed understanding of the maxillary sinus anatomy and variations before the SFE [9]. Maxillary sinus septa increase sinus membrane perforation during SFE [5]. It is one of the most prevalent maxillary sinus anatomical variations, with a prevalence ranging from 9 to 70% in patients and 13–58% in sinuses according to the diagnostic method [5, 10–13].

Sinus septa etiology remains unclear, and some studies have reported more maxillary sinus septa in edentulous than dentate subjects [7, 8]. It has been hypothesized that after tooth loss, edentulous maxillary sinuses may have additional septa due to pneumatization [14]. Previous studies have shown that panoramic radiographs are unreliable for identifying sinus septa [15]. Some authors showed that panoramic radiographs provide a mistaken diagnosis between 11.8% and 52.6% of the time compared to Computed Tomography (CT) [11]. Recent guidelines of the European Association of Osseointegration (EAO) and the International Team for Implantology (ITI) recommend preoperative Three-Dimensional (3D) imaging techniques, such as CT and CBCT imaging of the maxillary sinus and adjacent anatomical structures, to prevent SFE complications [14, 16]. It is prudent to use CBCT when evaluating maxillary sinuses as one of the greatest benefits is reducing the patient’s radiation exposure and cost [4, 6]. Before the surgical approach for SFE, it is essential to understand the locations and morphology of septa on CBCT scans to minimize membrane perforation [5, 14].

Since this study focused on Yemeni population, it is worth mentioning that habitual khat chewing in Yemen has a detrimental effect on oral and periodontal health status, which can cause pocket depth, gingival recession, and teeth loss on the chewing side among khat chewers [17–20]. Nevertheless, the correlation between khat chewing and the failure rate of dental implants has not yet been examined in the literature. Further, to our knowledge, no study has yet reported the CBCT-based investigation of sinus septa among Yemeni populations. The purpose of this study was to determine the prevalence and morphological variations of maxillary sinus septa, as well as their influence by age, gender, and dental status, in addition to the effect on the sinus membrane pathology. The null hypothesis was that no association between the maxillary sinus septa and gender, age, or dental status, as well as the sinus septa have no effect on sinus membrane pathology.

## Materials and methods

### Study Design and patients selection

This is a cross-sectional study conducted on 880 sinuses using CBCT images of 440 Yemeni patients from Sana’a and Taiz cities, the top largest population cities in The Republic of Yemen. The randomly selected CBCT images of the maxilla were picked up from stored data in the hospital system during the period from September 2019 to December 2021. All measurements were taken twice by the same observer at an interval of 8 weeks. The CBCT images were made for routine dental treatments. The institution’s policy includes patients’ consent to participate in any trial and approvals of using their data with a full explanation of the benefits and risks of any procedure.

The protocol of the study was approved by the Ethics Committee of Taiz University, Faculty of medicine and health science (ECTU2022/2–5). Moreover, all methods were carried out in accordance with the principles of the declaration of Helsinki.

#### Inclusion criteria

The patients of this study must be aged 18 years or above as well his/ her CBCT image of the complete bilateral maxillary sinuses was clear with good quality.

#### Exclusion criteria

The patient who met at least one of the following criteria was excluded; the patient whose age was less than 18 years; that because the growth of his/ her sinus was not completed yet, patients with a history of dental trauma or maxillary sinus surgery or dental implants or congenital diseases as cleft palate or experienced developmental anomaly in the domain of the maxillary sinus. The incomplete sinus CBCT image due to a small field of view (FOV) and the poor quality of CBCT images or artifacts due to various causes (beam hardening, noise, metal, and ring artifact) were also excluded.

### Data Collection

#### Sociodemographic characterizations

The sociodemographic characterizations, including the age and gender of the patients who met the above inclusion criteria, were collected at the time of CBCT scanning and then recruited to investigate their anticipated effect on the septa parameters.

#### CBCT images acquisition and investigation

**Fig. 1:**
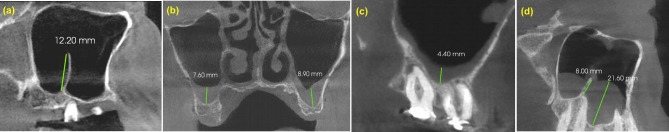
(**a**) Determination the septum height on the sagittal section, (**b**) Coronal view of sinus membrane thickness measurement in edentulous sinus ridge, (**c**) sagittal view of membrane thickness measurement in the dentate ridge and (**d**) measurement of both septum height and membrane thickness in pathological maxillary sinus as unhealthy sinus membrane

**Fig. 2:**
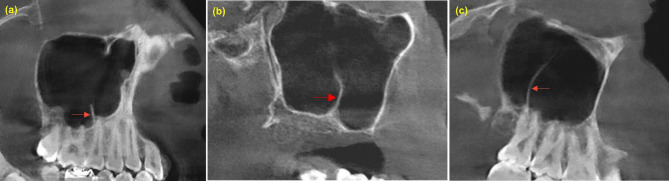
Sagittal CBCT sections showing several septa locations (**a**) anterior, (**b**) middle, (**c**) posterior

**Fig. 3:**
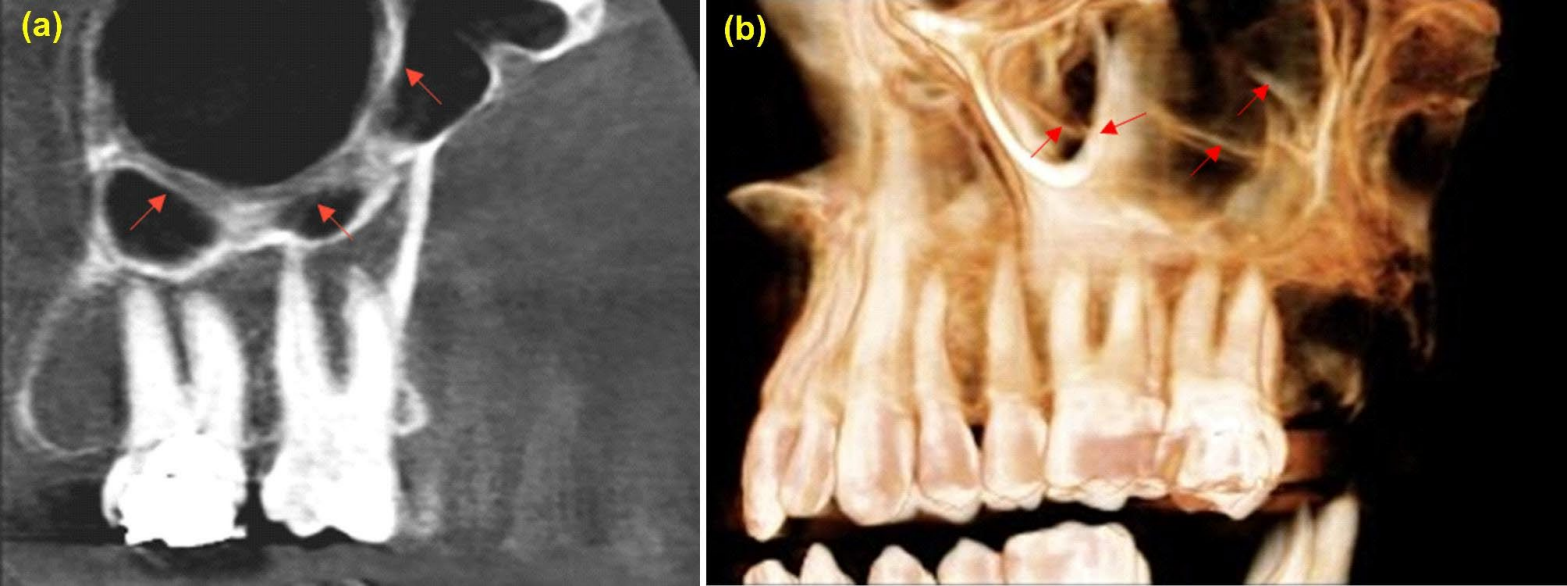
(**a**) Sagittal section of multiple and complete sinus septa (**b**) 3D view of maxillary sinus septa

**Fig. 4:**
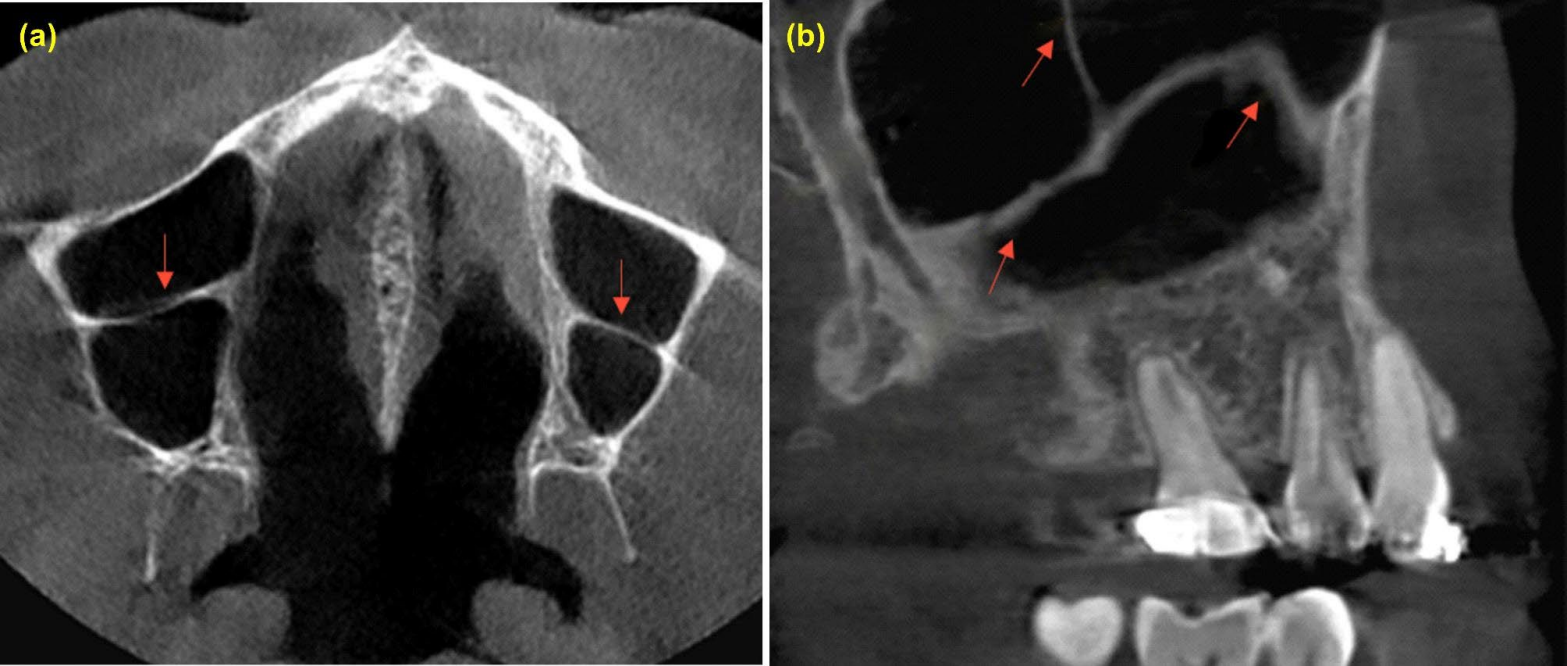
(**a**) axial section of bilateral complete septa (**b**) sagittal section of multiple and complete septa with coronal and sagittal orientation

Vatech, Pax-i3D Green™ machine was used for CBCT imaging with 50–90 KVp and 4–16 mA for 7.2 s. Bilateral maxillary sinuses of all selected patients were fully observed and evaluated. Invivo Anatomage (version 6) was utilized to determine and/ or calculate the morphometric characters of the maxillary sinus septa (numbers, configurations, heights, locations, orientations, and morphology) and thickness of Schneiderian membrane The prevalence of maxillary sinus septs was determined in each sinus and patient on the sagittal, coronal, and axial sections of CBCT images. The threshold of septa height is at least 2.5 mm on the sagittal section by drawing a line from the base of septa to the highest point Fig. 1(a). Further, on both sagittal and coronal sections, the membrane thickening was measured by drawing a line from the base of sinus to the highest point of the membrane Fig. 1(b, c), maxillary sinus pathology as healthy or unhealthy based on thickening of the sinus mucosa, and the cutoff point that more than 3 mm Fig. 1(d). The location was determined in accordance with the presence or absence of teeth into the anterior: in the region corresponding to premolars, middle: in the region above first and second molars and posterior: in the region distal to the second molar Fig. 2. A sinus with more than one septum was considered multiple septa, and when extended from one wall to another wall named complete septa Fig. 3. Septa presence in both sides were known as bilateral sinus septa with the orientation either sagittal, coronal or axial Fig. 4.

### Statistical analysis

Statistical Package for Social Sciences (SPSS) software (version 24, IBM Corp.) was employed for data entry and analysis. A descriptive statistic for the studied variables was calculated. The Effect of Gender, Age, and Dental Status on the Septa was estimated by Chi square test. The normal distribution (Kolmogorov-Smirnov test) of the related variables were initially identified to specify either parametric or nonparametric tests will be applied. The influence of septa on sinus membrane pathology was estimated by Chi square test. The *P* value < 0.05 was significantly considered.

## Results

### Sociodemographic characterizations

More than 2000 CBCT images of the maxilla were initially evaluated using the Invivo Anatomage program (version 6). The included CBCT images of 440 patients for 880 maxillary sinuses (approximately 22%) were fit to this study according to the mentioned inclusion and exclusion criteria above and hence were selected for further evaluation. The sociodemographic and related variables of the selected patients are presented in Table 1. Among 440 patients, 259 (58.9%) were females, and 181 (41.1%) were males. The patient’s age was divided into four groups; less than 24 years old group (25%), 25–34 years old group (25.2%), 35–44 years old group (18.2%), and more than 45 years old group (31.6%). The septa were found in the maxillary right sinus of 69 (15.7%) of the study patients, 79 (18%) in left sinus, and 133 (30.2%) bilaterally. Overall, the septa were found in 63.9% (281/440) of patients and 47% (414/880) of sinus segments. The average height of the sinus septa was 5.18 ± 2.23 mm as shown in Table 2. The maximum number of septa in a single sinus was higher in the left (5 septa) compared to the right (4 septa). As shown in Table S1, 70.9% (624/880) of the maxillary sinus membrane in both sides were healthy with the sinus membrane thickness was 3 mm or less. 63.8% (561/880) of sinuses were fully dentate while 22.1(195/880) were partially dentate and 14.1% (124/880) were complete edentulous sinus ridges.

### Configurations, locations, Origins, and orientations of Septa

The common configuration of septa was complete on both maxillary sides; 154 (27.5%) on the right and 172 (30.7%) on the left as a complete versus 112 (20%) and 122 (21.8%) as right and left partial septa, respectively Table S2. The results showed that both septa configuration (complete and partial) were found more on the left than on the right side.

It is clear that most of the septa were located in the middle of both maxillary sides, 117 (20.9%) on the right side and 124 (22.1%) on the left side, (43%) in both sides. However, 103 (18.4%) and 112 (20%) of septa were located in the anterior of the right and left, respectively, and (38.4%) in both sides. The posterior contained the least numbers of septa; 46 (8.2%) in the right and 58 (10.4%) in the left, and (18.6%) in both sides. According to the origin, the numerous septa originated from floor; 155 (27.7%) on the right side and 150 (26.8%) on the left side, and (54.5%) in both sides.

The most frequent of the septa orientation was coronal; 180 (32.1%) on the right side and 190 (33.9%) on the left side, and (66%) in both sides, followed by sagittal; 85 (15.2%) on the right side and 103 (18.4%) in the left side. The axial orientation is the least since only one septum for each side was found.

**Table 1:** Socio-demographic characterizations of the study patients

Variable		Frequency	Percent %
**Gender**			
	Male	181	41.1
	Female	259	58.9
**Age**			
	Less than 25	110	25.0
	25–34	111	25.2
	35–44	80	18.2
	≥ 45	139	31.6
**Presence of Septa**			
	None	159	36.1
	Right	69	15.7
	Left	79	18.0
	Both Sides	133	30.2
**Sinus Membrane Pathology**			
	Healthy	624	70.9
	Unhealthy	256	29.1
**Dental Status**			
	Dentate	561	63.75
	Partially D.	195	22.1591
	Edentulous	124	14.0909

**Table 2:** Height of septa on both maxillary sides

Height of Septa (mm)		
Maxillary Right Side	Maxillary Left Side	Both Sides
Mean ± SD	Mean ± SD	Average Mean ± SD
5.04 ± 2.02	5.32 ± 2.45	5.18 ± 2.23

### Effect of the gender, Age, Sinus membrane Status, and Dental Status on the Septa

The effects of gender, age, sinus membrane status, and dental status on the presence of septa are individually presented in Table 3. The Chi square test results showed that gender and sinus membrane status exerted no significant effect on the septa (P = 0.226 and 0.354, respectively). On the same line, the Chi square test results showed that age and dental status exerted no significant effect on the septa (P = 0.567 and 0.506, respectively). The number of septa in the maxillary right and left sides according to age, gender, sinus membrane status, and dental status are presented in Table S3.

**Table 3:** Effect of sociodemographic factors on the septa of the study patients

Variable (n)		Septa		P Value
		Present	Absent	
**Gender (440)**				
	Male (181)	122 (67.4)	59 (32.6)	0.226
	Female (259)	159 (61.4)	100 (38.6)	
**Age (440)**				
	Less than 25 (110)	74 (67.3)	36 (32.7)	0.567
	25–34 years (111)	74 (66.7)	37 (33.3)	
	35–44 (80)	50 (62.5)	30 (37.5)	
	≥ 45 (139)	83 (59.7)	56 (40.3)	
**Dental Status (880)**				
	Dentate (561)	351 (62.6)	210 (37.4)	0.506
	Partially D. (195)	131 (67.2)	64 (32.8)	
	Edentulous (124)	80 (64.5)	44 (35.5)	
**Sinus Membrane Pathology (880)**				
	Healthy (624)	405 (64.9)	219 (35.1)	0.354
	Unhealthy (256)	157 (61.3)	99 (38.7)	

### Effect of the Presence and absence of Septa on the Sinus membrane Pathology

The mean thickness of the maxillary right side membranes in the presence and absence of septa was 4.74 ± 1.60 mm and 4.96 ± 2.22 mm, respectively Table 4; which showed no significant difference (P = 0.789). The mean thickness of the maxillary left side membranes in the presence and absence of septa were 5.14 ± 2.40 mm and 5.11 ± 2.52 mm, respectively, which showed no significant difference (P = 0.806).

**Table 4:** Thickness of membrane in the maxillary right and left sides according to presence and absence of septa

Maxillary Right Side				Maxillary Left Side			
Variable		Thickness of membrane (mm)	P Value	Variable		Thickness of membrane (mm)	P Value
		Mean ± SD				Mean ± SD	
**Septa**	Present	4.74 ± 1.60	0.789	**Septa**	Present	5.14 ± 2.40	0.806
	Absent	4.96 ± 2.22			Absent	5.11 ± 2.52	

## Discussion

### Sinus septa variations and morphology

The present study findings found maxillary sinus septa in about two third of the patients (63.9%), and half of the sinuses exhibited septa (47%), which was noteworthy and equivalent to the literature and consistent with previous studies that showed a prevalence ranging from 9 to 70% in patients and 13–58% in sinuses [5, 10–12, 16]. In the literature, the prevalence of maxillary sinus septa varies significantly between studies of various nations and ethnicities. China, Korea, Brazil, Iran, Saudi Arabia, Turkey, and South Africa reported rates of 46.9%, 22.93%, 27%, 44,4%, 44.8%, 45.9%, 47.6%, and 69%, respectively [4, 21–27]. The sample size, criteria of septa (2–4 mm height), races and nations, and imaging modalities (CBCT, CT, panoramic, surgical observation, cadavers) could influence the different septa prevalence rates [21].

The septa were more prevalent unilaterally 33.7% (left 18% and right 15.7%) compared to bilateral (30.2%) in both sinuses. The maximum number of septa in a single sinus was higher in the left (5 septa) compared to the right (4 septa). Further, the septa were more prevalent in males than females, which is consistent with most previously published studies [4, 11]. However, Park et al. found the prevalence of septa to be higher among females [28].

In the present study, most septa were located on the floor with middle position and coronal orientation. About 43% were located in the middle region, 38.4% was located in the anterior region, and 18.6% were located in the posterior region. The most common orientations of the septa were coronal (66%), followed by sagittal (33.6%), and axial (0.4%) directions. The present study is in line with the previous studies that reported a majority of septa were oriented in the coronal/buccopalatal /transverse direction in a range of 61.8–87.6% [29].

The presence of maxillary sinus septa was significantly higher in the middle region in both the right and left sinuses, agreeing with systematic review studies that found the septa were positioned in the middle region [5, 11, 12, 30, 31], corresponding to first and second molars [22, 31] in the range between 35.7 and 70.94% and divided the sinus into two or more sections with a coronal orientation. However, some authors have demonstrated a higher rate of septa coincidence in the posterior molars [2, 3]. Nevertheless, our results are inconsistent with some authors who found that the highest rate of maxillary septa prevalence was located anteriorly [7, 32].

These results reveal the clinical importance for implant surgical procedures. A higher number of septa were observed in the first and second molar (middle) region, with coronal orientation, since the first and second molar regions are among those with the most bone loss after tooth extraction and often necessitating SFE treatments. According to classification system from Irinakis et al., the coronal orientation represents a lower risk of membrane perforation as Class I septa orientation increases the predictability of treatment unless it has a combination with other orientations as in Class IV [33].

In the present study, the average height of the septa of the maxillary right and left sides were 5.04 and 5.32 mm, respectively, with the entire mean of 5.6 mm, which was consistent with the septa average height of the previous studies in the range of 5.4–7.3 mm [4, 28, 29].

### Effect of the gender, Age, and Dental Status on the Septa

The present study indicated no significant correlation between the prevalence of septa and patient age or gender, which is consistent with prior studies [9, 24, 30, 34, 35]. Other studies showed a negative correlation only between septa and gender [2, 22, 36–38].

The present study found no relationship between dental status (dentate, partially edentulous, edentulous) and the prevalence of septa (63.8% of dentate, 22.1% of partially edentulous, and 14.1% of edentulous). This finding is consistent with Shen et al. [22] who observed no correlation between septa prevalence and the absence of maxillary first or second molars, consistent with recent studies [2, 14, 21, 24, 38, 39]. The present study contradicts other studies [35, 40, 41] which showed edentulous ridges have more septa than dentate ridges, as concluded by Krennmair et al. and Kim et al., who proposed the hypothesis that atrophic edentulous maxilla considers secondary septa [42, 43].

Primary/congenital and secondary/acquired classification of maxillary sinus septa has limited clinical significance since septa can appear anywhere in the maxillary sinus and do not emerge as a result of sinus pneumatization following tooth loss [14]. The etiology of septa formation is unknown, but genetics may affect septa formation during middle-face region development. Hence, maxillary sinus septa should be classified based on their location, orientation, and morphology since they are clinically significant during SFE procedures. Septa cause 11–56% of sinus membrane perforations during SFE [14, 24, 44].

### Effect of the Presence and absence of Septa on the Sinus membrane Pathology

The prevalence of mucosal thickening ranged between 35.1 and 66%, and thickening is considered normal between 1 and 3 mm [13]. The perforation rate was higher in thicker (≥ 2 mm) and thinner membranes (< 1 mm) according to Lin YH et al. [45]. In the present study, the reference for maxillary sinus pathology as healthy or unhealthy was Bronstein et al.[44] who diagnosed the pathology of maxillary sinus according to thickening of the sinus mucosa and the cutoff point of more than 3 mm, beyond which mucosal thickening of the maxillary sinus is regarded as pathological, we followed Ata Ali et al. and Vogiatzi et al.[13, 46].

The present study found no significant correlation between Sinus Membrane Pathology and the presence or absence of septa on both the right and left sides (P = 0.789 and P = 0.806), respectively. Findings from N. Kocak et al. in 2019 matched our results and observed no significant difference (P = 0.863) between septa presence and maximum membrane mucosal thickness in 376 patients and 500 sinuses [24]. By contrast, Cakur et al. reported that sinus septa might cause the Schneiderian membrane’s thinness, as membrane thickness is inversely related to sinus septa [47]. However, Rancitelli et al. found no positive correlation between septa height and membrane thickness. The thickness of the membrane at the septa origin is almost twice as thick as it is away from the septa origin [48].

The present study to the best of our knowledge, is the first study that is focused on the relation between maxillary sinus septa and maxillary sinus pathology (healthy and unhealthy), unlike the previous studies that studied the relation between sinus septa and sinus membrane in the position of septa. The present study has common limitation that is a cross-sectional evaluation of CBCT images from only two cities; thus, the findings cannot be generalized to all populations and the measurements matched by single observer. Therefore, future studies are strongly recommended to confirm the present findings.

## Conclusion

Based on our findings, the prevalence of septa among Yemeni population were 63.9% in patients and 47% in sinuses. Many septa were completed (58.2%), located in the middle (43%), originated from the floor (54.5%), and oriented coronally (66%). It’s noteworthy to mention that the prevalence of septa was significant and equivalent to the highest recorded in the literature yet. The sociodemographic (gender and age) and dental status (dentate, partially dentate, and edentulous) do not affect the presence of septa, nor does the septa presence influence the sinus membrane pathology. Therefore, preoperative CBCT imaging of the maxillary sinus is recommended for safe dental implantation, particularly when sinus floor elevation is planned.

## Electronic supplementary material

Below is the link to the electronic supplementary material.

**Additional File 1: Table S1** Number of septa in the maxillary right side versus number of septa in the maxillary left side of all study subjects; **Table S2** Configuration, locations, origins and orientations of septa in the right and left sides; **Table S3** Numbers of septa according to age, gender, sinus membrane status, and dental status

## Data Availability

All datasets and materials used and analyzed during the current study are available from the corresponding author on reasonable request.

## Abbreviations

CBCT: Cone Beam Computed Tomography
SFE: Sinus Floor Elevation
CT: Computed Tomography
3D: Three-Dimensional
EAO: European Association of Osseointegration
ITI: International Team for Implantology
