# Supplementary material for: Cone-beam computed tomographic analysis of maxillary sinus septa among Yemeni population: a cross-sectional study
Source: BMC Oral Health. 2023 Jul 8;23:466. doi: 10.1186/s12903-023-03124-6 (PMC10329384; doi:10.1186/s12903-023-03124-6)
Supplement: Supplementary file 1 — Additional File 1: Table S1 Number of septa in the maxillary right side versus number of septa in the maxillary left side of all study subjects; Table S2 Configuration, locations, origins and orientations of septa in the right and left sides; Table S3 Numbers of septa according to age, gender, sinus membrane status, and dental status [file 12903_2023_3124_MOESM1_ESM.docx]

| Table S1 Number of septa in the maxillary right side versus number of septa in the maxillary left side of all study subjects | | | | | | |
| --- | --- | --- | --- | --- | --- | --- |
| **Number of septa in the maxillary right side** | | | | | | |
| **Number of septa in the maxillary left side** | N | **0** | **1** | **2** | **3** | **4** |
|  | **0** | 159 | 64 | 5 | 0 | 0 |
|  | **1** | 6 1 | 60 | 20 | 5 | 1 |
|  | **2** | 15 | 27 | 7 | 3 | 0 |
|  | **3** | 3 | 2 | 3 | 0 | 2 |
|  | **4** | 0 | 0 | 1 | 1 | 0 |
|  | **5** | 0 | 0 | 1 | 0 | 0 |

| **Table S2** Configuration, locations, origins and orientations of septa in the right and left sides | | | | | | | |
| --- | --- | --- | --- | --- | --- | --- | --- |
| **Variable** | | **Septa** | | | | | |
|  |  | **N** | **Right** | | **Left** | | **Both Sides** |
|  |  |  | **No. Patients** | **Total, n (%)** | **No. Patients** | **Total, n (%)** | **Total %** |
| **configuration** | | | | | | |  |
|  | Complete |  |  | **154, (27.5)** |  | **172, (30.7)** | **326, (58.2%)** |
|  |  | 1 | 106 | 106 | 111 | 111 | 217 |
|  |  | 2 | 21 | 42 | 23 | 46 | 88 |
|  |  | 3 | 2 | 6 | 2 | 6 | 12 |
|  |  | 4 |  |  | 1 | 4 | 4 |
|  |  | 5 |  |  | 1 | 5 | 5 |
|  | Partial |  |  | **112, (20.0)** |  | **122, (21.8)** | **234, (41.8%)** |
|  |  | 1 | 92 | 92 | 96 | 96 | 188 |
|  |  | 2 | 10 | 20 | 10 | 20 | 40 |
|  |  | 3 |  |  | 2 | 6 | 6 |
| **Location** | | | | | | | |
|  | Anterior |  |  | **103, (18.4)** |  | **112, (20.0)** | **215, (38.4%)** |
|  |  | 1 | 83 | 83 | 88 | 88 | 171 |
|  |  | 2 | 10 | 20 | 10 | 20 | 40 |
|  |  | 4 |  |  | 1 | 4 | 4 |
|  | Middle |  |  | **117, (20.9)** |  | **124, (22.1)** | **241, (43%)** |
|  |  | 1 | 107 | 107 | 106 | 106 | 213 |
|  |  | 2 | 5 | 10 | 9 | 18 | 28 |
|  | Posterior |  |  | **46, (8.2)** |  | **58, (10.4)** | **104, (18.6%)** |
|  |  | 1 | 42 | 42 | 58 | 58 | 100 |
|  |  | 2 | 2 | 4 |  |  | 4 |
| **Origin** | | | | | | | |
|  | Floor |  |  | **150, (26.8)** |  | **155, (27.7)** | **305, (54.5%)** |
|  |  | 1 | 126 | 126 | 128 | 128 | 254 |
|  |  | 2 | 9 | 18 | 9 | 18 | 36 |
|  |  | 3 | 2 | 6 | 3 | 9 | 15 |
|  | Roof |  |  | **85, (15.2)** |  | **89, (15.9)** | **174, (31.1%)** |
|  |  | 1 | 63 | 63 | 73 | 73 | 136 |
|  |  | 2 | 11 | 22 | 8 | 16 | 38 |
|  | Lateral |  |  | **1, (0.2)** |  | **4, (0.7)** | **5, (0.9%)** |
|  |  | 1 | 1 | 1 | 4 | 4 | 5 |
|  | Median |  |  | **5, (0.9)** |  | **4, (0.7)** | **9, (1.6%)** |
|  |  | 1 | 5 | 5 | 5 | 4 | 9 |
|  | Anterior Wall |  |  | **20, (3.5)** |  | **28, (5)** | **48, (8.5%)** |
|  |  | 1 | 20 | 20 | 19 | 19 | 39 |
|  |  | 2 |  |  | 3 | 6 | 6 |
|  |  | 3 |  |  | 1 | 3 | 3 |
|  | Posterior Wall |  |  | **5, (0.9)** |  | **14, (2.5)** | **19, (3.4%)** |
|  |  | 1 | 5 | 5 | 14 | 14 | 19 |
| **Orientation** | | | | | | | |
|  | Coronal |  |  | **180, (32.1)** |  | **190, (33.9)** | **370, (66%)** |
|  |  | 1 | 128 | 128 | 122 | 122 | 250 |
|  |  | 2 | 17 | 34 | 28 | 56 | 90 |
|  |  | 3 | 6 | 18 | 4 | 12 | 30 |
|  | Sagittal |  |  | **85, (15.2)** |  | **103, (18.4)** | **188, (33.6%)** |
|  |  | 1 | 64 | 64 | 78 | 78 | 142 |
|  |  | 2 | 9 | 18 | 11 | 22 | 40 |
|  |  | 3 | 1 | 3 | 1 | 3 | 6 |
|  | Axial |  |  | **1, (0.2)** |  | **1, (0.2)** | **2, (0.4%)** |
|  |  | 1 | 1 | 1 | 1 | 1 | 2 |

| **Table S3** Numbers of septa according to age, gender, sinus membrane status, and dental status | | | | | | | | | | | | | | |
| --- | --- | --- | --- | --- | --- | --- | --- | --- | --- | --- | --- | --- | --- | --- |
| **Variable** | | **No. Septa** | | | | | | | | | | | | |
|  |  | **Right** | | | | **Left** | | | | | | | | |
|  |  | **1** | **2** | **3** | **4** | **1** | | **2** | | **3** | | **4** | | **5** |
| **Gender** | | | | | | | | | | | | | | |
|  | Male | 64 | 17 | 4 | 3 | 57 | 28 | | 7 | | 0 | | 1 | |
|  | Female | 89 | 20 | 5 | 0 | 90 | 24 | | 3 | | 2 | | 0 | |
| **Age** | | | | | | | | | | | | | | |
|  | Less than 25 | 41 | 9 | 1 | 1 | 32 | 15 | | 3 | | 0 | | 0 | |
|  | 25-34 | 41 | 9 | 4 | 0 | 45 | 13 | | 1 | | 2 | | 1 | |
|  | 35-44 | 25 | 9 | 2 | 1 | 27 | 6 | | 4 | | 0 | | 0 | |
|  | ≥ 45 | 46 | 10 | 2 | 1 | 43 | 18 | | 2 | | 0 | | 0 | |
| **Dental Status** | | | | | | | | | | | | | | |
|  | Dentate | 90 | 23 | 7 | 2 | 97 | 33 | | 6 | | 2 | | 1 | |
|  | Partially D. | 42 | 11 | 2 | 1 | 31 | 7 | | 1 | | 0 | | 0 | |
|  | Edentulous | 21 | 3 | 0 | 0 | 19 | 12 | | 3 | | 0 | | 0 | |
| **Sinus Membrane Pathology** | | | | | | | | | | | | | | |
|  | Healthy | 109 | 26 | 7 | 3 | 105 | 36 | | 7 | | 2 | | 1 | |
|  | Unhealthy | 44 | 11 | 2 | 0 | 42 | 16 | | 3 | | 0 | | 0 | |
